# Multiplexing Iduronate-2-Sulphatase (MPS-II) into a 7-Plex Lysosomal Storage Disorder MS/MS Assay Using Cold-Induced Phase Separation

**DOI:** 10.3390/ijns9020032

**Published:** 2023-06-08

**Authors:** Elya Courtney, C. Austin Pickens, Carla Cuthbert, Konstantinos Petritis

**Affiliations:** Centers for Disease Control and Prevention, Atlanta, GA 30341, USA; ecourtney2@cdc.gov (E.C.); ogh6@cdc.gov (C.A.P.); ijz6@cdc.gov (C.C.)

**Keywords:** newborn screening, lysosomal storage disorder, Hunter syndrome, MPS-II, cold induced phase separation

## Abstract

Mucopolysaccharidosis type II (MPS-II, Hunter syndrome, OMIM:30990) is a lysosomal storage disorder (LSD) that results in iduronate 2-sulphatase (I2S) enzyme deficiency. MPS-II was added to the Recommended Uniform Screening Panel (RUSP) in August 2022; thus, there is an increased demand for multiplexing I2S into existing LSD screening assays. After incubation with LSD synthetic substrates, extracts are cleaned using liquid–liquid extraction with ethyl acetate or protein precipitation using acetonitrile (ACN). We investigated cold-induced water ACN phase separation (CIPS) to improve the combination of 6-plex and I2S extracts to create a 7-plex assay, and compared it to room temperature ACN and ethyl acetate liquid–liquid extraction. The extracts were dried and resuspended in the mobile phase, and then analyzed using an optimized 1.9 min injection-to-injection liquid chromatography method coupled with tandem mass spectrometry (LC-MS/MS). The combination of ACN and CIPS improved the detection for I2S products without significant detriment to other analytes, which is attributable to a more complete coagulation and separation of heme, proteins, and extracted residual salts. Using CIPS for sample cleanup in dried blood spots (DBS) appears to represent a promising and straightforward way of achieving cleaner sample extracts in a new 7-plex LSD screening panel.

## 1. Introduction

Mucopolysaccharidosis type II (MPS-II, Hunter syndrome, OMIM:30990) is a lysosomal storage disorder (LSD) that results in iduronate 2-sulphatase (I2S) enzyme deficiency. MPS-II was added to the Recommended Uniform Screening Panel (RUSP) in August 2022; thus, there is an increased demand to multiplex I2S in existing LSD screening assays. LSD newborn screening is mandated by many states in the United States. Although there are dozens of LSDs, current assays often include Pompe disease (added to RUSP in 2013) and mucopolysaccharidosis type I (MPS-I, added to RUSP in 2018), with many laboratories working towards the inclusion of MPS-II. These disorders all stem from insufficient lysosomal enzymatic activity, leading to a build-up of macromolecules that are not properly catabolized. This results in the inappropriate accumulation and storage of complex carbohydrates, glycosaminoglycans, and lipids [1].

Current LSD platforms in newborn screening include fluorescence-based methods and tandem mass spectrometry (MS/MS) with sample introduction using flow injection analysis (FIA) or liquid chromatography (LC). Researchers have attempted to create a 7-plex assay (i.e., screening for seven LSDs in parallel using MS/MS in a single assay); however, structural similarity between substrates for I2S and alpha-L-iduronidase (IDUA) results in the cross-reactivity of the I2S substrate with IDUA. Therefore, producing a true 7-plex assay that is capable of screening all disorders from a single dried blood spot (DBS) punch is challenging. Instead, current methods focus on I2S as a stand-alone assay (1-plex), and extracts from 6-plex assays (i.e., MPS-I, Pompe, Gaucher, Fabry, Krabbe, and Niemann–Pick A/B disorders) and a 1-plex I2S assay are combined downstream after the enzymes are inhibited. (2) Following the punching of DBS, incubation takes place in an aqueous, highly buffered solution using non-volatile salts that extract high amounts of proteins. This extract is not compatible with downstream liquid chromatography–mass spectrometry (LC-MS/MS) analysis, so an additional cleanup step is required. There are currently two approaches used. The most common uses liquid–liquid extraction with ethyl acetate (EtOAc), which quenches the reaction and eliminates salts and proteins [2]. The second approach uses acetonitrile (ACN) at room temperature (RT-ACN) for quenching and protein precipitation, with the extract from the top of the sample wells being transferred for analysis after centrifugation [3,4,5]. Both approaches have drawbacks. The water:EtOAc liquid–liquid extraction method leads to a very clean extract, but suffers from the low recovery of analytes of interest, such as enzymatic products and internal standards (IS). ACN addition alone effectively precipitates the proteins and recovers more product, but recovers a high amount of salts that can be detrimental to the robustness of the LC-MS/MS assay.

**Figure 1:**
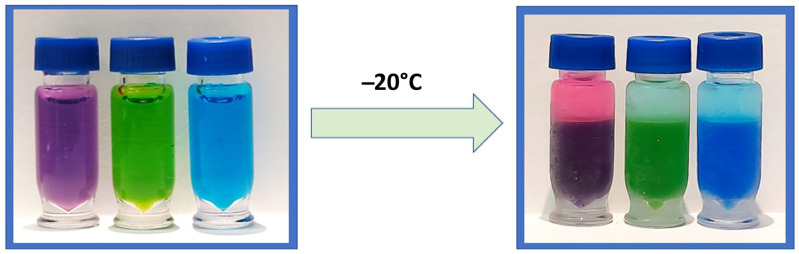
A before-and-after visual of CIPS using an ACN and water mixture containing dye to demonstrate clear phase separation after freezing at −20 °C.

Miscible mixtures of water with ACN can be phase-separated using either kosmotropic salts or by decreasing the temperature to around −17 °C, the latter being coined as cold-induced aqueous ACN phase separation (CIPS) [6]. CIPS causes true phase layers to form (Figure 1), creating a liquid–liquid extraction that separates unwanted materials from the desired sample. This performs the same function as the layers formed by the EtOAc method, but reduces the method’s overall complexity and the risk posed to the analyst from EtOAc. This study investigates the use of CIPS to improve the combination of 6-plex and 1-plex extracts to increase the recovery of analytes of interest and eliminate non-volatile salts, while improving the robustness and throughput of the assay. To our knowledge, this is the first reported use of CIPS in a newborn screening assay.

## 2. Materials and Methods

All product information and sources (i.e., solvents, consumables, etc.) are presented in Supplemental Table S1. The DBS analyzed for this study were produced using a base pool of double leuko-depleted blood and three enriched levels using cord blood to produce 0, 5, 50, and 100% relative enzyme activity pools. Samples were 3.2 mm DBS punches taken from LSD quality control (QC) materials (Lot 2108), from levels A (0%) through to D (100%). The enzyme substrates, internal standards (S&IS), and buffers were purchased from PerkinElmer^®^ as LSD 6-plex S&ISs and 1-plex I2S S&ISs. A list of enzymes and quantification *m/z* ions is available in Table 1.

6-plex and 1-plex S&ISs were resuspended using 21 mL of the corresponding buffer per vial. Two identical 96-well plates were prepared for each sample run, with 40 µL of 6-plex S&IS per well in one and 40 µL of 1-plex S&IS per well in the other. The plates were heat-sealed with foil, centrifuged for 30 s to ensure DBS coverage, and incubated at 37 °C for 18 h. RT-ACN quenching steps and EtOAc washing were performed, according to previously reported methods [2,4].

For the CIPS ACN quench, 200 µL of −20 °C ACN was added to each sample well in both the 6-plex and 1-plex plates. The solution was mixed 10 times with a pipette, and the plate was resealed with foil, centrifuged for 1 min at 3500 rpm, and placed in a −20 °C freezer for 12 min. In this study, all CIPS sample upper layers were transferred within 2 min of removal from the freezer. The low temperature caused the ACN and aqueous contents to separate, forming an ACN-enriched upper layer containing the enzyme products and an aqueous-enriched bottom layer containing excess salts, heme, and proteins. After removing both plates (i.e., 6-plex and 1-plex) from the freezer, 75 µL was transferred from the ACN layer to a fresh plate and combined with corresponding sample extracts from the other plate, creating a total volume 150 uL of extracts from both plates in a new 96-well plate. The combined extracts were dried for 10 min under N2 gas at 50 °C with flow rates of 50 L/min upper and 20 L/min lower, and then resuspended in 150 µL of 30% ACN, 70% water, and a 0.1% formic acid solution. The samples were shaken at RT for 10 min in a sealed plate prior to injection.

**Figure 2:**
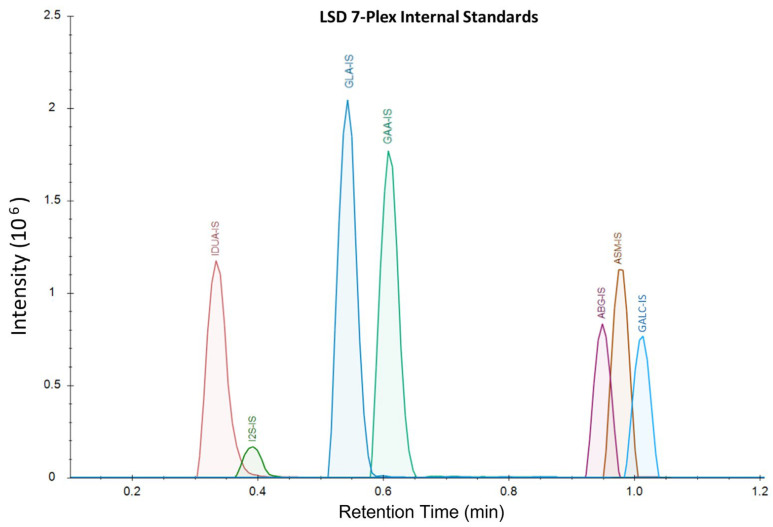
LSD 7-plex IS extracted ion currents, displaying retention times and intensities for each analyte. The corresponding product peaks co-elute with IS when running DBS samples. Substrates undergoing in-source fragmentation to products have distinct and separate retention times and signals from the IS and true products.

The samples were analyzed on a Waters™ Xevo TQD using LC-MS/MS with a Acquity™ XSelect CSH 1.7 µm × 2.1 mm × 50 mm UPLC column and the corresponding 5 mm guard column. Mobile Phase A was comprised of 20% ACN, 80% water, and 0.01% formic acid. Mobile Phase B was comprised of 70% ACN, 30% methanol, and 0.01% formic acid. Weak wash was comprised of 50% water and 50% ACN, and strong wash was 100% ACN. The LC-MS gradient ran from 75%A:25%B to 1%A:99%B from 0 to 1.10 min at a flow rate of 0.790 mL/min, with all analytes eluting by 1.2 min (Figure 2). The column was washed for 15 s before injection and 25 s after injection. The column was heated at 60 °C during the analysis. The method’s total turnaround time for each sample was 1.9 min.

Enzyme activity is typically calculated using the following equation:

(1) $$Activity\;\left(\frac{\mu M}{hr}\right)=\frac{M_{1}}{M_{2}}\times \left[IS\right]\times \frac{V_{IS}}{V_{DBS}}\times \frac{1}{T}$$

where *M*_1_ is the peak area of the product, *M*_2_ is the peak area of the IS, [*IS*] is the concentration of the IS solution for the analyte, *V_IS_* is the volume of the IS solution added at incubation (40 µL), *V_DBS_* is the estimated volume of blood contained in the DBS punch (3.1 µL), and *T* is the time of incubation [5]. Note that IS solutions were prepared at a different concentration for this assay than what is typically standard. We presented the effects of CIPS on *M*_2_ (from Equation (1)) to demonstrate how IS recoveries are significantly impacted by sample workup. Since ISs are isotopically labeled versions of the enzymatic products, we chose to present only IS data for brevity purposes. Enzyme products demonstrated similar phase affinities. The IS data from the DBS-extracted samples were used to account for matrix effects during the comparison stage. The raw MS data were imported into Waters™ TargetLynx (V4.2) to obtain the peak areas, and were imported into the open-source software Skyline (V 20.1.0.155) for visualization purposes.

## 3. Results

**Figure 3:**
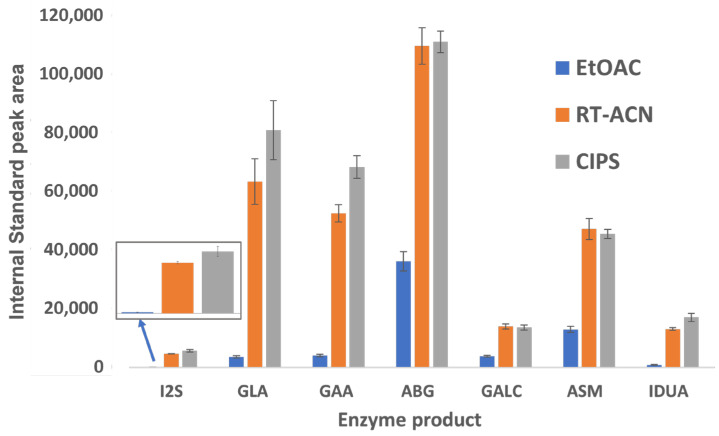
The recovery of enzymatic product ISs using three different cleanup steps. The data are displayed as peak areas ± standard deviations for each enzyme product, shown for each sample workup condition. The IS peak areas were extracted from the C2108 DBS sample data. I2S and IDUA IS peak areas with EtOAc extraction were notably low.

The peak areas for all IS and analytes were significantly higher for RT-ACN and CIPS methods compared to the EtOAc method (Figure 3). For I2S and IDUA, peak areas for the IS and product were below 1000 counts when using EtOAc, and slightly higher with the CIPS method than with RT-ACN. GLA and GAA also showed a strong affinity for ACN-based separations, with slight increases in peak areas with CIPS and comparable product recoveries.

## 4. Discussion

Further method optimization is in progress for multiple method steps. CIPS requires the samples to be at low temperatures during the organic phase separation process, and since fewer samples imply lower thermal capacity, the time it takes for the samples to warm is related to the number of samples on the 96-well plate. Using two minutes as the time to transfer in this study was determined to be a safe option in order to reduce potential plate-to-plate variability in our laboratory. This time may vary with ambient temperatures, the number of samples on each plate, and handling procedures. The use of a cold base plate chiller upon removing CIPS samples from the freezer can extend the time buffer for the top layer transfer. Continued minor adjustments to the chromatographic method will be made to improve analyte peak resolution and separation.

The recommended S&IS concentrations from the flow injection analysis (FIA)-MS/MS LSD 6-plex assay are higher than those required for this LC-MS assay. The chromatographic separation of enzyme products causes less ion suppression, improving the assay’s sensitivity. Reducing the concentration of S&IS for this assay led to improved peak shapes and baseline resolution for analytes that need to be separated. While the method separates the substrate from the product in all cases, there is an observable risk of column saturation and loss of baseline resolution between these peaks when using higher concentrations of S&IS. Based on the aforementioned issues and preliminary data from our pilot studies, we used the provided buffer to resuspend S&IS vials at 21 mL instead of at 3.3 mL. We observed sufficient signals for all analytes at this concentration with improved peak shapes.

Our study improves upon the existing methods for sample preparation and LC-MS/MS analysis for LSD biomarkers. The product and IS recovery are 2.5–60 times higher with CIPS than with EtOAc (Figure 3), depending on the enzyme. CIPS requires fewer handling steps and smaller wash volumes, reducing the potential for operator errors in pipette mixing and transfer steps, along with product loss. There is a distinct benefit to adding CIPS to ACN methods, and analyte recovery is comparable or slightly improved for all analytes between the CIPS and RT-ACN methods. Since the separation of heme, proteins, and excess aqueous layer salts is more complete with CIPS than with RT-ACN alone, the robustness of the assay is improved. This method also reduces the time required to analyze each sample when compared to previously published methods that can screen for MPS II and is similar to FIA methods [2,5,7]. This LC-MS/MS-based LSD 7-plex assay is a high-throughput and cost-effective method to add screening for MPS-II to the newborn screening panel.

## Figures and Tables

**Table 1:** A list of LSDs and their respective enzymes, for which activities are measured in this 7-plex assay. Enzyme product parents and MS/MS product ions are also shown.

Disease	Enzyme	Enzyme Product Parent Ion (*m/z*)	MS/MS Product Ion (*m/z*)
MPS-I	IDUA—alpha-L-iduronidase	431.3	322.1
Fabry	GLA—alpha-galactosidase A	489.3	389.3
Pompe	GAA—acid alpha-glucosidase	503.3	403.3
Gaucher	ABG—acid beta-glucosidase	391.4	271.3
Krabbe	GALC—galactoceramidase	417.3	264.2
Niemann–Pick	ASM—acid sphingomyelinase	405.4	264.2
MPS-II	I2S—iduronate-2-sulphatase	649.3	364.3

## Data Availability

The data relevant to this study are presented in this article. Requests for additional data can be directed to the corresponding author.

## Supplementary Materials

The following supporting information can be downloaded at: https://www.mdpi.com/article/10.3390/ijns9020032/s1, Supplemental Table S1. List of products with vendor information and product numbers for relevant chemicals, materials, equipment, and software.

## References

[1] D’Avanzo F., Rigon L., Zanetti A., Tomanin R. (2020). Mucopolysaccharidosis Type II: One Hundred Years of Research, Diagnosis, and Treatment. Int. J. Mol. Sci..

[2] Elliott S., Buroker N., Cournoyer J.J., Potier A.M., Trometer J.D., Elbin C., Schermer M.J., Kantola J., Boyce A., Turecek F. (2016). Pilot study of newborn screening for six lysosomal storage diseases using Tandem Mass Spectrometry. Mol. Genet. Metab..

[3] Burton B.K., Hoganson G.E., Fleischer J., Grange D.K., Braddock S.R., Hickey R., Hitchins L., Groepper D., Christensen K.M., Kirby A. (2019). Population-Based Newborn Screening for Mucopolysaccharidosis Type II in Illinois: The First Year Experience. J. Pediatr..

[4] Burton B.K., Charrow J., Hoganson G.E., Waggoner D., Tinkle B., Braddock S.R., Schneider M., Grange D.K., Nash C., Shryock H. (2017). Newborn Screening for Lysosomal Storage Disorders in Illinois: The Initial 15-Month Experience. J. Pediatr..

[5] Spacil Z., Tatipaka H., Barcenas M., Scott C.R., Turecek F., Gelb M.H. (2013). High-Throughput Assay of 9 Lysosomal Enzymes for Newborn Screening. Clin. Chem..

[6] Shao G., Agar J., Giese R.W. (2017). Cold-induced aqueous ACN phase separation: A salt-free way to begin quick, easy, cheap, effective, rugged, safe. J. Chromatogr. A.

[7] Scott C.R., Elliott S., Hong X.Y., Huang J.Y., Kumar A.B., Yi F., Pendem N., Chennamaneni N.K., Gelb M.H. (2020). Newborn Screening for Mucopolysaccharidoses: Results of a Pilot Study with 100,000 Dried Blood Spots. J. Pediatr..

