# Supplementary material for: Multiplexing Iduronate-2-Sulphatase (MPS-II) into a 7-Plex Lysosomal Storage Disorder MS/MS Assay Using Cold-Induced Phase Separation
Source: Int J Neonatal Screen. 2023 Jun 8;9(2):32. doi: 10.3390/ijns9020032 (PMC10298912; doi:10.3390/ijns9020032)
Supplement: Supplementary file 1 [file IJNS-09-00032-s001.zip › IJNS-2335396-SI.pdf]

**Multiplexing Iduronate-2-Sulphatase (MPS-II) into a 7-Plex Lysosomal Storage Disorder MS/MS Assay Using Cold-Induced Phase Separation**

**Elya Courtney [1], C. Austin Pickens, Carla Cuthbert, and Konstantinos Petritis***

[1] Centers for Disease Control and Prevention; ECourtney2@cdc.gov
* Correspondence: K. Petritis, kpetritis@cdc.gov

**Supplementary Material**

| Product | Vendor | Location | Product Number |
|---|---|---|---|
| 6-Plex S&IS | PerkinElmer® | Hopkinton, MA, USA | CCM05458Y000EA<br>Lot#: 20210902 |
| I2S S&IS | PerkinElmer® | Hopkinton, MA, USA | 4400-0010<br>Lot#: 20210802 |
| 96-well plate | VWR | Radnor, PA, USA | 9444-104 |
| LiChrosolv® EtOAc | VWR | Radnor, PA, USA | 1.03649.1000 |
| Optima LC/MS Water | Fisher | Waltham, MA, USA | W6-4 |
| Optima LC/MS Acetonitrile | Fisher | Waltham, MA, USA | A955-4 |
| Optima LC/MS Methanol | Fisher | Waltham, MA, USA | A456-4 |
| Foil Seals | Biosero | San Diego, CA, USA | STA3100006 |
| LC/MS Formic Acid | Thermo Scientific | Waltham, MA, USA | 85178 |
| XSelect CSH UPLC Column | Waters™ | Milford, MA, USA | 186005296 |
| XSelect CSH Guard Column | Waters™ | Milford, MA, USA | 186005303 |
| TargetLynx | Waters™ | Milford, MA, USA | Version 4.2 |
| Skyline - open source | Seattle Quant | Seattle, WA, USA | V 20.1.0.155 |
| Centrifuge – Tina 380R | Hettich | Tuttlingen, Germany | 1706-01 |
| Incubator Shaker – MaxQ 4450 | Thermo Scientific | Waltham, MA, USA | No longer sold |
| N2 Plate Dryer – SPE Dry 96 Dual | Biotage® | Charlotte, NC, USA | SD2-9600-DHS-NA |

Supplement Table S1: List of products with vendor information and product numbers for relevant chemicals, materials, equipment, and software.
